# Influence of leftover antibiotics on self-medication in Saudi Arabia: a cross-sectional study

**DOI:** 10.12688/f1000research.130364.1

**Published:** 2023-03-20

**Authors:** Bader Al-Mehmadi, Saad Alsubaie, Omar Al-Morikhi, Fawaz Alqahtani, Waad Almutairi, Maryam Al-Mutairi, Mohammed Alotaibi, Saud Alenazi, Khalid Alanazi

**Affiliations:** 1 Assistant Professor of Medicine, Rheumatology Consultant, Department of Internal Medicine, College of Medicine, Majmaah University, Al-Majmaah, 11952, Saudi Arabia; 2 College of Medicine, Majmaah University, Al-Majmaah, Saudi Arabia, Al-Majmaah, Saudi Arabia

**Keywords:** Leftover antibiotics, self-medication, antimicrobial resistance

## Abstract

**Background:** Antimicrobial resistance is increasing at an alarming rate. The use of antibiotics without a prescription by  a patient or other family members and their inappropriate storage have caused serious health issues as it would lead to antibiotic resistance and exposure to risk of harmful adverse effects unnecessarily. Exploring causes behind their storage in homes and reuse will help us identify the problem in depth and help in recommending effective solutions.

**Methods:** This is a cross-sectional study. Our target study population was the residents of Saudi Arabia. Data were collected by an online questionnaire and analysed by SPSS.

**Results**: A total of 738 participants answered the online questionnaire, from all ages, genders, nationalities, and different socioeconomic backgrounds residing in different regions across the kingdom of Saudi Arabia. 76.42% knew that an antibiotic is a chemical substance used to treat infections. The participants were questioned about when do they start using antibiotics, to which 95.66% (n=706) responded, after consulting a physician, 3.25% (n=24) said when they felt ill for any reason, and 1.08% (n=8) replied after first attempting herbal medicine. 147 participants admitted that they store excess pills of antibiotics after being prescribed for an infection and re-use them later on for symptoms like sore throat and fever.

**Conclusions:** Although strict measures have been put into effect from the Saudi Ministry of Health to reduce antimicrobial resistance caused by misuse of antibiotics by restricting the dispense of antibiotics from pharmacies without a medical prescription, a large portion of the population regardless of age, level of education, or professional background, have continued to store excess pills of antibiotics after an infection treatment and re-use them once they think they need them for new symptoms. This advises for further revision of the current measures to fill those gaps and reduce this habit.

## Introduction

Recently, an alarming issue in human health has come to light: the misuse of unused antibiotics. Antibiotics are potent drugs used to treat bacterial infections, but improper administration or storage can cause bacteria to develop resistance. This is why it is essential to complete the entire course of any prescribed antibiotic. Inadequate use of antibiotics can eventually lead to the development of antibiotic-resistant bacteria, so it is also important to be aware of the risks associated with stockpiling antibiotics. ^1^ The primary risk associated with the misuse of unused antibiotics is the development of drug-resistant bacteria. If bacteria are repeatedly exposed to a particular antibiotic, they will develop resistance over time. In addition, if left untreated, this could lead to additional, more difficult diseases that may also be resistant to other drugs, ultimately leaving the infected significantly worse off than before they took the antibiotics. ^2^

Antibiotic resistance can frequently be traced directly to leftover or unfinished medications that have been hidden in homes or sold illegally online without a valid prescription. To combat this issue, it is crucial that governments around the world implement appropriate regulation systems so that appropriate safety precautions are taken whenever possible. ^1^ In a study on the knowledge, attitude, and practice of antibiotic use and misuse among adults attending Primary Healthcare Centres (PHCs) in Arar, several factors have been proposed to explain the increase in antibiotic resistance, including self-medication, cultural factors, behavioral factors, lack of health education, socioeconomic status, education level, and patients‘/parents' pressure on physicians and pharmacists. In this study, 479 applicants participated, and the knowledge score (62.6±25.6) regarding the safe use of antibiotics was relatively high. ^3^

A similar study involving 141 participants in the city of Buraidah and the use of antibiotics found that 75.2% of the participants believe that antibiotics can be used to treat influenza and the common cold, 46.1% believe it is beneficial for fever, and 22.2% believe it is beneficial for headache. Additionally, 30% of participants believed that antibiotics should be kept at home and used when a family member becomes ill. In addition, 67% of study participants stated that antibiotic resistance is only an issue for those who take it and has no bearing on others. ^4^ A second study conducted in Kuwait with 680 participants revealed that 187 (27.5%) of the study population had taken antibiotics without a doctor's prescription within the past year. The majority of them, 166 (88.8%), were also prescribed antibiotics during this period. Ninety-seven (51.9%) of the self-medicated respondents indicated that they had given an antibiotic to another person without a doctor's prescription. 119 (63.6%) reported using antibiotics that were initially prescribed for an infection that recurred, and 21 (11.2%) for a different type of infection. In Kuwait, 59 (31.6% of respondents who self-medicated) purchased antibiotics directly from private pharmacies. Family members (n=50; 26.7%) and friends (n=7; 3.5%) were also sources of antibiotics. ^5^ A study was conducted to determine the prevalence of antibiotic self-medication in Saudi Arabia. More than one-third of respondents (43.4%) indicated that they sometimes self-medicate with antibiotics. Tonsillitis and pharyngitis (76.7%) are the most frequently reported conditions for which antibiotics were self-prescribed. They also discovered that the most common source of antibiotic self-medication was a previous prescription (36.6%). ^6^ Likewise, a study was conducted to evaluate the knowledge and attitudes of Saudi citizens regarding antibiotic use and self-medication. 63.6% of participants reported purchasing antibiotics from pharmacies without a prescription, and 71.1% reported not finishing the antibiotic course because they felt better. In addition, 44.7% of those who used antibiotics (prescribed or non-prescribed) reported keeping antibiotics from incomplete courses of treatment for future use. ^7^ Another study evaluates the general population's knowledge and attitudes regarding antibiotic use in Jeddah, Saudi Arabia. Almost half of those who used antibiotics did so without a prescription, obtaining them from a retail pharmacy (63.9%), a private clinic (15.3%), or someone else's supply (20%). In addition, they discovered that the majority of antibiotic prescriptions were for fever, pain, and/or inflammation (58.2%), followed by respiratory illnesses (21.2%). ^8^ According to a study conducted in China, 48.1% of participants kept antibiotics at home for their children. Regarding the origins of antibiotics, 63.1% of participants who kept antibiotics at home obtained them through a previous prescription, whereas 35.3% reported purchasing their antibiotics from pharmacies. ^9^ For the factors associated with keeping antibiotics at home, the study found that those with a higher level of education were more likely to do so, while those with a medical background were more likely to do so. ^9^

In a study conducted in Saudi Arabia measuring the knowledge of health-related students about antibiotics, 50.0% of participants believed antibiotics could be used safely without consulting a physician. ^10^ The majority of participants in the same study believed that antibiotics can be used instead of anti-inflammatory drugs to treat pain and inflammation. ^10^ In the previous study, the majority of participants believed that antibiotics could be used to treat viral infections, ^10^ and a similar study conducted in Qatar found that nearly half of participants believed antibiotics could be used to treat viral illness. ^11^ This study also found that 82% of participants used antibiotics without a prescription, 37% used antibiotics prescribed for another family member, and 27% used antibiotics prescribed to them for a similar condition. ^11^

According to research conducted in Saudi Arabia, certain risk factors may be more prevalent in Saudi Arabia than in other countries. As presented by a Riyadh hospital, one of the risk factors is the misuse of antibiotics. Unoptimized antibiotic dosage is another risk factor for the development of bacterial resistance. In addition, one of the most significant risk factors is travel to Saudi Arabia's holy cities, which presents an opportunity for the spread of infectious diseases. ^12^ Another study that examined the use of antibiotics in tertiary hospital settings found that factors such as bed occupancy rate, average length of stay, and number of admissions had no bearing on the development of antimicrobial resistance (AMR), and the same was true for the number of bacteria and percentage of isolates in the group. ^13^ In a study examining participants' knowledge and attitudes regarding antibiotics, it was discovered that between 20 and 50% of the study population shared leftover antibiotics with family members. ^14^ In a separate study, the researchers discovered that a relative's leftover antibiotics, the use of leftover antibiotics from a similar episode of disease, and the presence of a runny nose, sore throat, and fever were all examples of situations in which leftover antibiotics were administered. ^15^

In 2022, 231 parents participated in a cross-sectional study conducted in six rural primary health centers in Peru to investigate the causes of limited awareness and self-medication of children with antibiotics. The largest knowledge gap was observed among 183 parents (79%) who were unaware that antibiotics are ineffective against viral infections. More than half of the parents (n=120, 52%) admitted giving their children antibiotics without a doctor's prescription. ^16^

From September to December of 2018, a cross-sectional survey of medical students from three universities in Colombia was conducted. 532 medical students participated in the study. In the past year, 49.1% of individuals utilized antibiotics. Only 18.2% of those surveyed were familiar with the term “antimicrobial stewardship,” and only 69.3% knew that empiric antibiotic therapy contributes to antibiotic resistance. 11.6% believe that antibiotics should be discontinued as soon as the patient's symptoms improve, while 24.6% believe that prescribing broad-spectrum antibiotics is the most effective way to ensure that the patient is treated. In terms of their actions, 28.5% of respondents are aware that resistance is a multifaceted problem, but they take no action because they believe that individual actions will have little impact. ^17^

Similarly, another cross-sectional study was conducted to investigate rural population knowledge, attitudes, and practices regarding self-medication in south-western Saudi Arabia in 2019. 58% of the 500 responses were from women, while 42% were from men. Pain (38.1%), influenza (26.3%), cough (24%), and allergies (24%) were treated with self-medication. 85% of patients did not consult a physician, were unaware of medicine information inserts, and did not check the expiration date. ^18^

## Methods

### Study design and study area

The study design was a cross-sectional online survey, ^22^ and was distributed through social media platforms to the residents of Saudi Arabia in different regions. A total of 738 participants were included in the study.

### Study time period

The study was conducted after the ethical approval was taken. The data collection was started on 29 July 2022 and completed on 31 December 2022.

### Target population

The study included participants that were currently residing in Saudi Arabia; male and female both were included, and those who were ≥18 years of age. All others who were not willing to participate, did not give consent or <18 years of age were excluded.

### Patient consent

Informed consent was the part of online questionnaire and only participants who were voluntarily willing to participate filled the questionnaire.

### Sample size and sampling technique

As this electronic survey will be conducted in all regions of Saudi Arabia, a cluster sampling technique was used to collect the data from 738 participants. The minimum required sample size of 700 was calculated using the level of precision formula by placing the following values

$$n=\frac{Z^{2}\times p\times q}{d^{2}\times \mathrm{DE}},$$

where (Z=1.96, p=0.50, q=0.50, d=0.05, DE =2).

### Instrument of data collection

A self-prepared questionnaire was used to collect data from the participants. The questionnaire had three sections. Section one contained questions about the demographic data, section two contained questions about the knowledge of antibiotics, and section three contained questions about solutions.

### Data analysis

The data was entered and analyzed using SPSS 26.0 (v.26.0, IBM Corporation, New York, USA) (RRID:SCR_002865). Mean and standard deviation will be given for quantitative variables. Frequencies and percentages were given for qualitative variables. Knowledge scores were calculated by counting the correct answers, which were then converted to percentage to see whether the participants had poor, good or excellent knowledge. Pearson-Chi-Squared/Fisher Exact tests were applied to observe associations between qualitative variables. A p-value of <0.05 will be considered as statistically significant.

## Results

738 participants of all ages, genders, nationalities, and socioeconomic backgrounds, residing in various regions of the kingdom of Saudi Arabia, responded to the online questionnaire (see Table 1). ^21^ 76.42% (n=564) knew what an antibiotic was a chemical substance used to treat infections, 14.09% (n=104) thought it was used to relieve pain, 3.25% (n=24) thought it was used to reduce fever, and 6.23% (n=46) had no idea. When asked when they begin using antibiotics, 95.66% (n=706) of the participants responded, after consulting a physician, 3.25 % (n=24) said when they felt ill for any reason, and 1.08% (n=8) replied after first attempting herbal medicine. When asked for which symptoms people most frequently use antibiotics, the following responses were provided: 38.08% (n=281) reported a sore throat, 32.11 (n=237) a fever, 27.10% (n=200) a cold, and 2.71% (n=20) a cough. 28.32% (n=209) of participants selected allergy as the most common side effect of antibiotics, followed by 21.82% (n=161) who selected stomachache, 7.87% (n=58) who selected rash, and 5.01% (n=37) who selected difficulty breathing. The remaining participants chose multiple adverse effects. When asked to define antibiotic resistance, 68.43% (n=505) of respondents said it is a microorganism's adaptability against antibiotics, 3.6% (n=27) said it is a decrease in antibiotic efficacy due to manufacturing errors, and 27.19% (n=206) said they had no idea.

**Table 1.** Demographics and characteristics of study participants.

Variable	n (%)
**Preferred Answering Language**	
**Arabic**	707 (95.8%)
**English**	31 (4.2%)
**Age**	
**Less than 20**	85 (11.52%)
**20-30**	377 (51.08%)
**30-40**	131 (17.5%)
**More than 40**	145 (19.65%)
**Marital Status**	
**Single**	448 (60.7%)
**Married**	273 (36.99%)
**Divorced**	11 (1.49%)
**Widow**	6 (0.81%)
**Do you have children?**	
**Yes**	256 (34.69%)
**No**	482 (65.31%)
**Number of children**	
**1 or 2**	74 (25.52%)
**3 or 4**	82 (28.28%)
**More than 4**	100 (34.48%)
**Nationality**	
**Saudi**	700 (94.85%)
**Non-Saudi**	38 (5.51%)
**Place of Residence**	
**Central region**	566 (76.69%)
**Eastern region**	67 (9.08%)
**Western region**	52 (7.05%)
**Northern region**	17 (2.3%)
**Southern region**	36 (4.88%)
**Level of education**	
**Bachelor degree or higher**	549 (74.39%)
**High school degree or less**	189 (25.61%)
**Occupation**	
**Healthcare worker**	268 (36.31%)
**Non-healthcare worker**	470 (63.69%)
**Average monthly income**	
**< 10,000 Saudi Riyals (<2660 USD)**	427 (63.96%)
**≥ 10,000 Saudi Riyals (≥2660 USD)**	266 (36.04%)
**Do you have any chronic illness?**	
**Yes**	613 (83.06%)
**No**	125 (16.94%)

When asked if they take antibiotics when experiencing any new non-specific symptom, the following responses were given by the participants: 46.07% (n=340) strongly disagreed, 29.95% (n=221) disagreed, 12.47% (n=92) were neutral, 7.32% (n=54) agreed, and 4.2% (n=31) strongly agreed. 71.54% (n=528) of the participants strongly agreed, 20% (n=149) agreed, 4.61% (n=34) were neutral, 1.76% (n=13) disagreed, and 1.90% (n=14) strongly disagreed when asked if they only use antibiotics with a prescription. 71.41% (n=527) of participants responded with strong agreement to the question, 21% (n=155) with agreement, 4.61% (n=34) with a neutral response, 2.44% (n=18) with disagreement, and 0.54% (n=4) with strong disagreement. When asked if they stored the antibiotics according to the label's instructions, 91.19% (n=673) said yes, while 8.8% (n=65%) said no.

When asked if limiting antibiotic prescriptions was a good idea, 91.33% (674 of 674) of participants agreed, while 8.67% (n=64) disagreed. When asked whether prescribing antibiotics encourages their misuse, 82.79% of the respondents (n=611) said “yes,” while 17.21% said “no.” When asked what they believed could be done to reduce antibiotic resistance, 35.64 % (n=263) said antibiotics should only be taken when prescribed by a doctor, 18.56% (n=137) said living a healthy lifestyle would increase immunity, lower the risk of infection, and reduce the need for antibiotics, and 11.92% (n=88) said antibiotics should only be used in life-threatening situations. Only 20.05% (n=148) of the respondents have participated in an antibiotics campaign, but 88.08% (n=650) believe that antibiotics campaigns can alter community habits.

**Figure 1.**
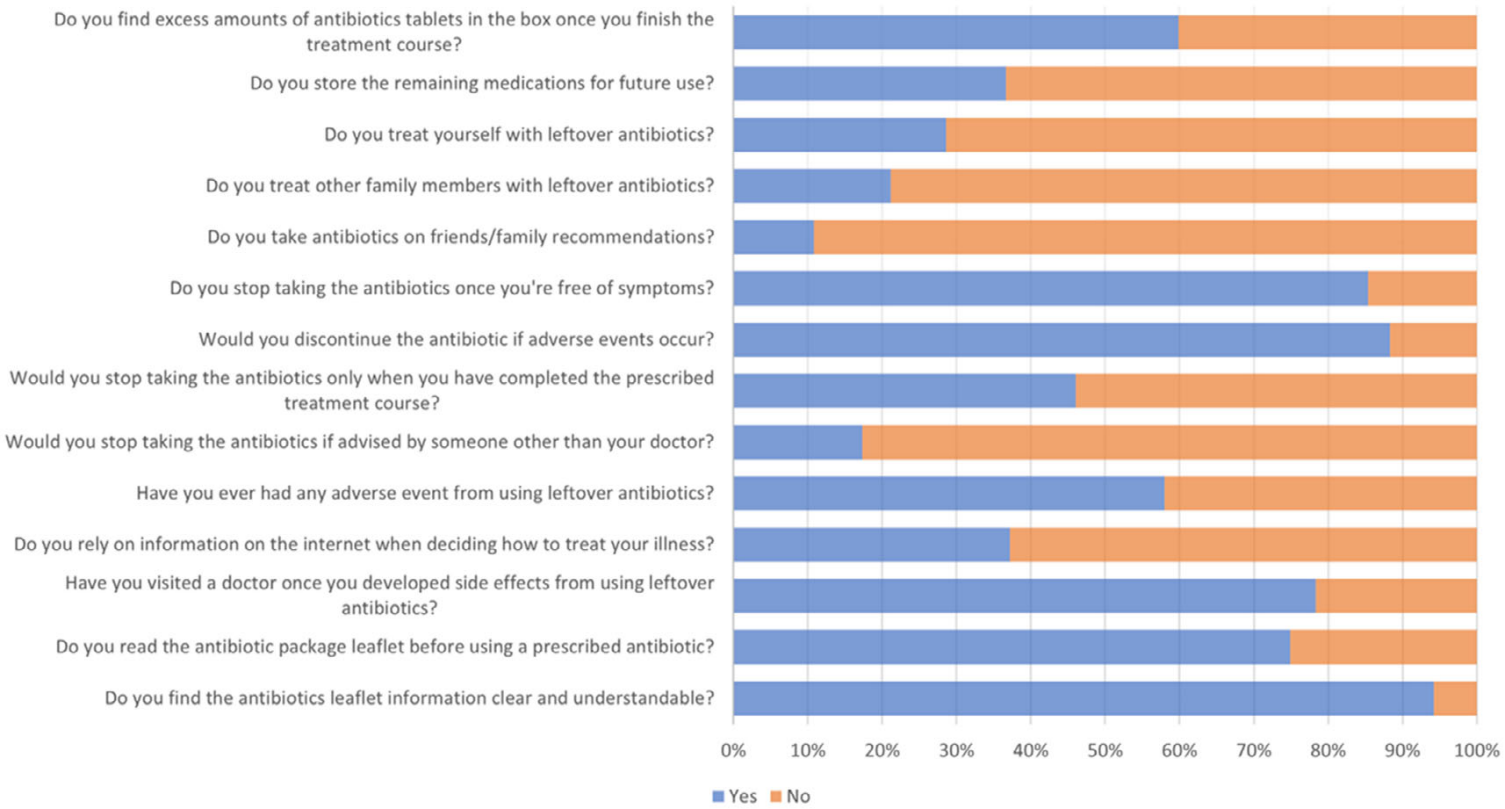
Knowledge about the Antibiotics.

There is no correlation between average household income and the storage and reuse of antibiotics (p>0.30). A statistically significant correlation existed between age and the use of unused antibiotics (p=0.0019). The remaining 311 participants did not use any leftover antibiotics: 33 (22.4%) were under 20 years of age, 77 (52.38%) were 20—29 years of age, 18 (12.24%) were 30—40 years of age, and 19 (12.93%) were older than 40 years of age. There was no statistically significant correlation between gender and the use of leftover antibiotics (p>0.05); among the 147 participants who used leftover antibiotics, 79 were male (53.74%) and 68 were female (46.26%). There was a statistically significant correlation between having children and reusing antibiotics; among the 147 participants who reused antibiotics, 30 (20.41%) had children, while 117 (79.59%) did not. There was no statistically significant correlation between the number of children and the use of leftover antibiotics. Eight (26.67%) of the 30 participants who used leftover antibiotics and had one or two children, nine (30%) had three or four children, and 13 (43.33%) had more than four children. There was no statistically significant correlation between the level of education and the use of unused antibiotics. Among the 147 participants who used leftover antibiotics, 102 (69.39%) had a college degree or higher, while 45 (30.61%) did not complete high school. There was no statistically significant correlation between occupation and the use of leftover antibiotics; 45 (30.61%) of the 147 participants who used leftover antibiotics were healthcare workers, while 102 (69.39%) were non-healthcare workers. We also found no statistical significance between chronic illness and knowledge of antibiotics or attitude toward unused antibiotics. Additional answers are depicted in Figure 1.

## Discussion

When asking what antibiotics are and when they should be used, having 738 participants from all regions of Saudi Arabia helped us gain a deeper understanding of the various backgrounds and educational levels of the participants. Providing an analysis of the impact of media and awareness campaigns, when asked when they typically begin using antibiotics, 95.66% (n=706) of respondents said after consulting with a physician. This majority may indicate a high level of awareness, but it may also indicate that the health ministry is effectively implementing and monitoring the restrictive system. Another study conducted in Saudi Arabia in 2021 revealed that 82% of participants who were able to obtain antibiotics did so without a prescription. ^10^

Another study conducted in Kuwait with 680 participants revealed that 27.5% of the study population had taken antibiotics without a prescription in the previous year. 97 (51.9%) of the self-medicated respondents reported giving an antibiotic to another individual without a prescription. 119 (63.6%) reported using antibiotics initially prescribed for a recurrent infection, while 21 (11.2%) reported using antibiotics for a different type of infection. Family members (n=50; 26.7%) and friends (n=7; 3.5%) were also sources of antibiotics. In Kuwait, 59 (31.6%) of respondents who self-medicated purchased antibiotics directly from private pharmacies (3). When asked when they first used antibiotics, 95.66% (n=706) of the participants in our study said after consulting a doctor, 3.25% (n=24) when they felt ill for any reason, and 1.08% (n=8) after trying herbal medicine for the first time. ^1^

A study revealed that 81.4% of the respondents believed that antibiotic resistance occurs when the body develops a resistance to antibiotics and ceases to function properly. ^19^ When asked what they believe could be done to decrease antibiotic resistance, 88.08% (n=650) of respondents believe that antibiotics campaigns can alter community habits. Community-wide attitude shifts can be easily implemented with the aid of campaigns, resulting in a decline in antibiotic resistance. There was a statistically significant correlation between participant age and the use of unused antibiotics (p=0.0019). 33 (22.4%) of the 147 participants who used unused antibiotics were younger than 20 years old, 77 (52.38%) were 20—29 years old, 18 (12.24%) were 30—40 years old, and 19 (12.93%) were older than 40 years old. We can attribute the increase in the use of leftover antibiotics in the 20—29 age group to multiple factors, including the high proportion of participants in this age group (51.08%). There were numerous perspectives on antibiotic use (n=377), resulting in unregulated use.

There was a statistically significant relationship between having children and reusing antibiotics; of the 147 participants who reused antibiotics, 30 (20.41%) had children, while 117 (79.59%) did not. This statistic indicates that participants with children were more hesitant than those not having any, either because they feared the side effects of reusing antibiotics or because they were more cautious when it came to their children. There was no statistically significant correlation between occupation and the use of leftover antibiotics; 45 (30.61%) of the 147 participants who used leftover antibiotics worked in healthcare, while 102 (69.39%) did not. This statistic indicates that although healthcare professionals have greater access to information about antibiotics, they continue to use unused antibiotics, which may contribute to an increase in antimicrobial resistance.

A 2022 cross-sectional study revealed that 79% of 183 parents were unaware that antibiotics are ineffective against viral illnesses. ^16^ 76.42% (n=564) of those surveyed in our study were aware that antibiotics are chemical substances used to treat bacterial infections; 14.09% (n=104) believed it was used to relieve pain; 3.25 % (n=24) believed it was used to reduce fever; and 6.23% (n=46) had no idea. A similar study involving 141 participants in the city of Buraidah and the use of antibiotics found that 75.2% of participants believed that antibiotics can be used to treat influenza and the common cold, 46.1% believed that antibiotics are beneficial for fever, and 22.2% believed that antibiotics are beneficial for headache. In addition, 30% of participants believed that antibiotics should be kept at home and administered to sick family members. In addition, 67% of study participants stated that antibiotic resistance is only a concern for those who take it and has no effect on others. ^15^ Antibiotics were used to treat pain (38.3%) and cough (24%) in another cross-sectional study conducted in south-western Saudi Arabia in 2019. According to our study, participants used antibiotics for the following reasons: 38.08% (n=281) reported a sore throat, 32.11 (n=237) reported a fever, 27.10% (n=200) reported a cold, and 2.71 (n=20) reported a cough.

When addressing the decision to stop antibiotics once the patient is symptom-free, studies have yielded variable results; however, 71.1% of patients did not complete their antibiotic treatment because they felt better. ^7^ A 2018 survey revealed that 11.6% of respondents believed that antibiotics should be discontinued as soon as symptoms subside. While our research yielded similar results to the first study, with 71.41% (n=527) of respondents stating that they complete the course as instructed; the first study had a larger sample size. ^18^ The presence of expired pharmaceuticals in the home can increase the risk of toxicity, suicide, and accidental poisoning of children, ^19^ whereas the presence of unused antibiotics indicates antibiotic misuse and may increase the likelihood of antibiotic resistance. ^20^

Storage at an improper temperature could have negative consequences. Low-stability medications, such as penicillin and cephalosporin, are easily weakened or rendered ineffective by improper storage temperature conditions. They may hinder the recovery from infections. In our study, 91.19% (n=673) of the participants stored the antibiotics in accordance with the label's instructions, while 8.81% (n=65) did not. Our study demonstrates that participants with a higher level of education keep and utilize unused antibiotics, corroborating a Chinese study which found that individuals with a higher level of education are more likely to keep antibiotics at home. In contrast to their findings, our study reveals that medical personnel were less likely to retain unused antibiotics than non-medical personnel. Among the 147 participants who used leftover antibiotics, 102 (69.39%) had a bachelor's degree or higher and 45 (30.61%) did not. 45 (30.61%) of the 147 participants who used leftover antibiotics were healthcare professionals, while 102 (69.39%) were not. ^9^

In a study, the prevalence of antibiotic self-medication in Saudi Arabia was determined. Over one-third of respondents, or 43.4% reported that they self-medicate with antibiotics on occasion. ^6^ While our study demonstrates that the majority of participants (71.54%; n = 528) only used antibiotics when prescribed, there were a number of participants who used antibiotics off-label. Tonsillitis and pharyngitis account for 76.7% of all instances of antibiotic self-medication. ^6^ Our study reveals that 38.08 percent (n=281) of individuals who self-medicate with leftover antibiotics do so for sore throat, 32.11% (n=237) for fever, 27.10% (n=200) for the common cold, and 2.71% (n=20) for cough. Another study assessed the knowledge and attitudes of the general population in Jeddah, Saudi Arabia, regarding the use of antibiotics. Nearly half of antibiotic users obtained them without a prescription from a retail pharmacy (63.9%), a private clinic (15.3%), or someone else's supply (20%). This study was published in 2018, prior to the 2019 announcement of penalties for violating Saudi Arabia's restrictive regulations. The regulations, which were announced for the first time in 2015, stipulated that pharmacists who sell antibiotics without a written prescription will be subject to a 100,000 SAR fine, license revocation, or six months in jail. In addition, they discovered that fever, pain, or inflammation were the most common reasons for taking antibiotics (58.2%), followed by respiratory illnesses (21.2%). ^8^ In a study conducted in Saudi Arabia to evaluate the knowledge of health science students regarding antibiotics, 50% of participants believed antibiotics could be used without consulting a doctor (8). 71.54% (n=528) of the participants in our study believed antibiotics should only be used when prescribed, with 48% (n=204) of these respondents being healthcare professionals.

## Conclusions

While it is understandable that people may believe storing unused antibiotics will be useful in the event of an emergency, there are numerous risks associated with doing so. Although the Saudi Ministry of Health has implemented stringent measures to reduce antimicrobial resistance caused by antibiotic misuse by restricting the dispensing of antibiotics from pharmacies without a prescription, a sizeable proportion (20%) of our sample participants, regardless of age, level of education, or profession, have continued to store excess antibiotic pills after an infection treatment and re-use them. This calls for further revision of the current measures to fill those gaps and reduce this practice, such as selling/distributing the exact number of pills required to treat the current infection per prescription and increasing awareness of the consequences of using antibiotics excessively without consulting a doctor. This may also have an economic benefit, as more antibiotic pills will be available for additional prescriptions.

## Ethical considerations

Ethical approval was received from the institutional review board of The Majmaah University for research committee (MUREC) (HA-01-R-088) with the ethical number MUREC-f uly.28/COM-2022/1O-2. Information from the questionnaire will be kept confidential and only used for statistical purposes.

## Data Availability

Due to confidentiality of our participants and our university ethical consideration, our data cannot be shared widely/openly. The datasets generated during and/or analyzed during the current study are not publicly available, but are available from the corresponding author on reasonable request after an Ethical Committee approval (Name: Bader Almehmadi, Assistant professor of medicine, rheumatology consultant ; email: b.almehmadi@mu.edu.sa; Article type Research article). The access to the data can be shared/granted for research process only and after the approval involving ethics committee and corresponding author. Figshare: Influence of leftover antibiotics on self-medication in Saudi Arabia أثر بقايا المضادات الحيوية في المنازل على سلوك المعالجة الذاتية في المملكة العربية السعودية .pdf. https://doi.org/10.6084/m9.figshare.22001894.v1 ^21^ This project contains the following extended data:
• Influence of leftover antibiotics on self-medication in Saudi Arabia أثر بقايا المضادات الحيوية في المنازل على سلوك المعالجة الذاتية في المملكة العربية السعودية .pdf (Questionnaire). Figshare: STROBE checklist for ‘Influence of leftover antibiotics on self-medication in Saudi Arabia: a cross-sectional study’. https://doi.org/10.6084/m9.figshare.22001603.v1. ^22^ Data are available under the terms of the Creative Commons Attribution 4.0 International license (CC-BY 4.0).
