# Knowledge and Attitude of self-medication with leftover antibiotics in Saudi Arabia: A cross-sectional study

**DOI:** 10.12688/f1000research.130364.2

**Published:** 2024-10-18

**Authors:** Bader Al-Mehmadi, Saad Alsubaie, Omar Al-Morikhi, Fawaz Alqahtani, Waad Almutairi, Maryam Al-Mutairi, Mohammed Alotaibi, Saud Alenazi, Khalid Alanazi

**Affiliations:** 1 Assistant Professor of Medicine, Rheumatology Consultant, Department of Internal Medicine, College of Medicine, Majmaah University, Al-Majmaah, 11952, Saudi Arabia; 2 College of Medicine, Majmaah University, Al-Majmaah, Saudi Arabia, Al-Majmaah, Saudi Arabia

**Keywords:** Leftover antibiotics, self-medication, antimicrobial resistance

## Abstract

**Background:**

Antimicrobial resistance is increasing at an alarming rate. The use of antibiotics without a prescription by a patient or other family members and their inappropriate storage have caused serious health issues as it would lead to antibiotic resistance and exposure to the risk of harmful adverse effects unnecessarily. Therefore, the study aimed to evaluate the current behaviour of antibiotic usage, storage, re-usage and misuse among the residents of Saudi Arabia.

**Methods:**

This is a cross-sectional study. Our target study population was the residents of Saudi Arabia. Data were collected by an online questionnaire and analysed by SPSS.

**Results:**

A total of 738 participants answered the online questionnaire from all ages, genders, nationalities, and socioeconomic backgrounds residing in different regions across the Kingdom of Saudi Arabia. 76.42% knew that an antibiotic is a chemical substance used to treat infections. The participants were questioned about when they started using antibiotics, to which 95.66% (n=706) responded after consulting a physician, 3.25% (n=24) said when they felt ill for any reason, and 1.08% (n=8) replied after first attempting herbal medicine. A total of 147 participants admitted that they store excess pills of antibiotics after being prescribed for an infection and reuse them later on for symptoms like sore throat and fever.

**Conclusions:**

The results indicated that nearly half of the participants used leftover antibiotics. Participants having children in their homes significantly reuse antibiotics. However, one-third of the participants didn’t complete the antibiotics course. A large portion of the population, regardless of age, level of education, or professional background, have continued to store leftover antibiotics after an infection treatment and reuse them once they think they need them for new symptoms. This advice further revises the current measures to fill those gaps and reduce this habit.

## Introduction

Recently, an alarming issue in human health has come to light: the misuse of leftover antibiotics. Antibiotics are potent drugs used to treat bacterial infections, but improper administration or storage can cause bacteria to develop resistance. ^1^ The primary risk associated with the misuse of leftover antibiotics is the development of drug-resistant bacteria. If bacteria are repeatedly exposed to a particular antibiotic, they will develop resistance over time. In addition, if left untreated, this could lead to additional, more difficult diseases that may also be resistant to other drugs, ultimately leaving the infected significantly worse off than before they took the antibiotics. ^2^

Antibiotic resistance can frequently be traced directly to leftover or unfinished medications that have been hidden in homes or sold illegally online without a valid prescription. To combat this issue, it is crucial that governments around the world implement appropriate regulation systems so that appropriate safety precautions are taken whenever possible. ^1^ Self-medication with antibiotics (SMA) is a prevalent manifestation of antibiotic misuse that can result in a number of harmful consequences, including antimicrobial-resistant bacteria. Antibiotic storage at home raises the risk of SMA.

For instance, a study reported that most of the participants believe that antibiotics can be used to treat influenza and the common cold, and antibiotics should be kept at home and used when a family member becomes ill. ^3^ Similarly a study conducted in Kuwait revealed that 50% of the self-medicated respondents indicated that they had given an antibiotic to another person without a doctor’s prescription. ^4^ A study was conducted to determine the prevalence of antibiotic self-medication in Saudi Arabia. More than one-third of respondents (43.4%) indicated that they sometimes self-medicate with antibiotics. The most common source of antibiotic self-medication was a previous prescription (36.6%). ^5^ According to a study conducted in China, 48.1% of participants kept antibiotics at home for their children. Regarding the origins of antibiotics, 63.1% of participants who kept antibiotics at home obtained them through a previous prescription, whereas 35.3% reported purchasing their antibiotics from pharmacies. ^6^

Likewise, a study conducted in Qatar found that nearly half of participants believed antibiotics could be used to treat viral illness. ^7^ This study also found that 82% of participants used antibiotics without a prescription, 37% used antibiotics prescribed for another family member, and 27% used antibiotics prescribed to them for a similar condition. ^7^

According to research conducted in Saudi Arabia, certain risk factors may be more prevalent in Saudi Arabia than in other countries. As presented by a Riyadh hospital, one of the risk factors is the misuse of antibiotics. Unoptimized antibiotic dosage is another risk factor for the development of bacterial resistance. In addition, one of the most significant risk factors is travel to Saudi Arabia’s holy cities, which presents an opportunity for the spread of infectious diseases. ^8^ Several factors have been linked to increase in antibiotic resistance, including self-medication, cultural factors, behavioral factors, lack of health education, socioeconomic. ^9^ The use of antibiotics without a prescription by a patient or other family members and their inappropriate storage have caused serious health issues as it would lead to antibiotic resistance and exposure to risk of harmful adverse effects unnecessarily. It is evident from the above literature that antimicrobial resistance and misuse of antibiotics has been increasing around the world. Studies conducted in Saudi Arabia about the use of antibiotics have been published around 3-8 years old. Furthermore, Saudi Ministry of Health (MOH) implemented a restriction policy for antibiotics use in 2018. ^10^ Therefore, it is necessary to evaluate the current behaviour of antibiotic usage, storage, re-usage and misuses among the residents of Saudi Arabia. Exploring the causes behind their storage in homes and reuse will help us identify the problem in depth and help in recommending effective solutions.

## Methods

### Study design and study area

The study design was a cross-sectional online survey, ^11^ and was distributed through different social media platforms to the residents of Saudi Arabia in different regions. A link to google form has been distributed through email, Facebook, and WhatsApp to the residents of Saudi Arabia. More than 1000 forms have been received. Missing information, incomplete forms and those who were less than 18 years were excluded and a total of 738 participants were included in the study.

### Study time period

The study was conducted after the ethical approval was taken. The data collection was started on 29 July 2022 and completed on 31 December 2022.

### Target population

The study included participants that were currently residing in Saudi Arabia; male and female both were included, and those who were ≥18 years of age.

### Sample size and sampling technique

As this electronic survey was conducted in all regions of Saudi Arabia, a cluster sampling technique was used to collect the data from 738 participants. The minimum required sample size of 700 was calculated using the level of precision formula by placing the following values

$$n=\frac{Z^{2}\times p\times q}{d^{2}\times \mathrm{DE}},$$

where (Z=1.96, p=0.50, q=0.50, d=0.05, DE =2).

### Instrument of data collection and patient consent 

A self-prepared questionnaire was used to collect data from the participants. The questionnaire was sent to expert to check its content validity. The questionnaire was then edited based on expert opinion. The final draft was face validated which was deemed reasonable. The questionnaire had three sections. Section one contained questions about the demographic data, section two contained questions about the knowledge of antibiotics, and section three contained questions about solutions. Informed consent was the part of online questionnaire and only participants who were voluntarily willing to participate filled the questionnaire.

### Data analysis

The data was entered and analyzed using SPSS 26.0 (v.26.0, IBM Corporation, New York, USA) (RRID:SCR_002865). Mean and standard deviation will be given for quantitative variables. Frequencies and percentages were given for qualitative variables. Knowledge scores were calculated by counting the correct answers, which were then converted to percentage to see whether the participants had poor, good or excellent knowledge. Pearson-Chi-Squared/Fisher Exact tests were applied to observe associations between qualitative variables. A p-value of <0.05 will be considered as statistically significant.

## Results

The participants of all ages, genders, nationalities, and socioeconomic backgrounds, residing in various regions of the Kingdom of Saudi Arabia participated in the study, and 738 responded to the online questionnaire (see Table 1). ^12^ Majority of the respondents (76.42%) knew that an antibiotic was a chemical substance used to treat infections, 14.09% (n=104) thought it was used to relieve pain, 3.25% (n=24) thought it was used to reduce fever, and 6.23% (n=46) had no idea. “When asked when they begin using antibiotics”, 95.66% (n=706) of the participants responded that “after consulting a physician”, whereas 3.25% and 1.08% practiced self-medication with reasons of onset of illness and after failed herbal treatment. When asked for which symptoms people most frequently use antibiotics, the following responses were provided: 38.08% (n=281) reported a sore throat, 32.11 (n=237) a fever, 27.10% (n=200) a cold, and 2.71% (n=20) a cough. Around less than one-third of the respondents, (28.32%) selected allergy as the most common side effect of antibiotics, followed by 21.82% (n=161) who selected stomachache, 7.87% (n=58) selected rash, and 5.01% (n=37) selected difficulty breathing. The remaining participants chose multiple adverse effects. When asked to define antibiotic resistance, 68.43% (n=505) of respondents said it is a microorganism’s adaptability against antibiotics, 3.6% (n=27) said it is a decrease in antibiotic efficacy due to manufacturing errors, and 27.19% (n=206) said they had no idea.

**Table 1.** Demographics and characteristics of study participants.

Variable	n (%)
**Preferred Answering Language**	
**Arabic**	707 (95.8%)
**English**	31 (4.2%)
**Age**	
**Less than 20**	85 (11.52%)
**20-30**	377 (51.08%)
**30-40**	131 (17.5%)
**More than 40**	145 (19.65%)
**Marital Status**	
**Single**	448 (60.7%)
**Married**	273 (36.99%)
**Divorced**	11 (1.49%)
**Widow**	6 (0.81%)
**Do you have children?**	
**Yes**	256 (34.69%)
**No**	482 (65.31%)
**Number of children**	
**1 or 2**	74 (25.52%)
**3 or 4**	82 (28.28%)
**More than 4**	100 (34.48%)
**Nationality**	
**Saudi**	700 (94.85%)
**Non-Saudi**	38 (5.51%)
**Place of Residence**	
**Central region**	566 (76.69%)
**Eastern region**	67 (9.08%)
**Western region**	52 (7.05%)
**Northern region**	17 (2.3%)
**Southern region**	36 (4.88%)
**Level of education**	
**Bachelor degree or higher**	549 (74.39%)
**High school degree or less**	189 (25.61%)
**Occupation**	
**Healthcare worker**	268 (36.31%)
**Non-healthcare worker**	470 (63.69%)
**Average monthly income**	
**< 10,000 Saudi Riyals (<2660 USD)**	427 (63.96%)
**≥ 10,000 Saudi Riyals (≥2660 USD)**	266 (36.04%)
**Do you have any chronic illness?**	
**Yes**	613 (83.06%)
**No**	125 (16.94%)

When asked if they take antibiotics when experiencing any new non-specific symptom, the following responses were given by the participants: 46.07% (n=340) strongly disagreed, 29.95% (n=221) disagreed, 12.47% (n=92) were neutral, 7.32% (n=54) agreed, and 4.2% (n=31) strongly agreed. When asked if they only use antibiotics with a prescription, 71.54% of the participants strongly agreed, 20% agreed, 4.61% were neutral, 1.76% disagreed, and 1.90% (n=14) strongly disagreed. The majority of participants, 71.41% (n=527) responded with strong agreement to the question if they complete the course of prescribed antibiotics, 21% (n=155) with agreement, 4.61% (n=34) with a neutral response, 2.44% (n=18) with disagreement, and 0.54% (n=4) with strong disagreement. When asked if they stored the antibiotics according to the label’s instructions, 91.19% (n=673) said yes, while 8.8% (n=65%) said no.

When asked if limiting antibiotic prescriptions was a good idea, 91.33% (674 of 674) of participants agreed, while 8.67% (n=64) disagreed. When asked whether prescribing antibiotics encourages their misuse, 82.79% of the respondents (n=611) said “yes,” while 17.21% said “no.” When asked what they believed could be done to reduce antibiotic resistance, 35.64 % (n=263) said antibiotics should only be taken when prescribed by a doctor, 18.56% (n=137) said living a healthy lifestyle would increase immunity, lower the risk of infection, and reduce the need for antibiotics, and 11.92% (n=88) said antibiotics should only be used in life-threatening situations. Only 20.05% (n=148) of the respondents have participated in an antibiotics campaign, but 88.08% (n=650) believe that antibiotics campaigns can alter community habits.

**Figure 1.**
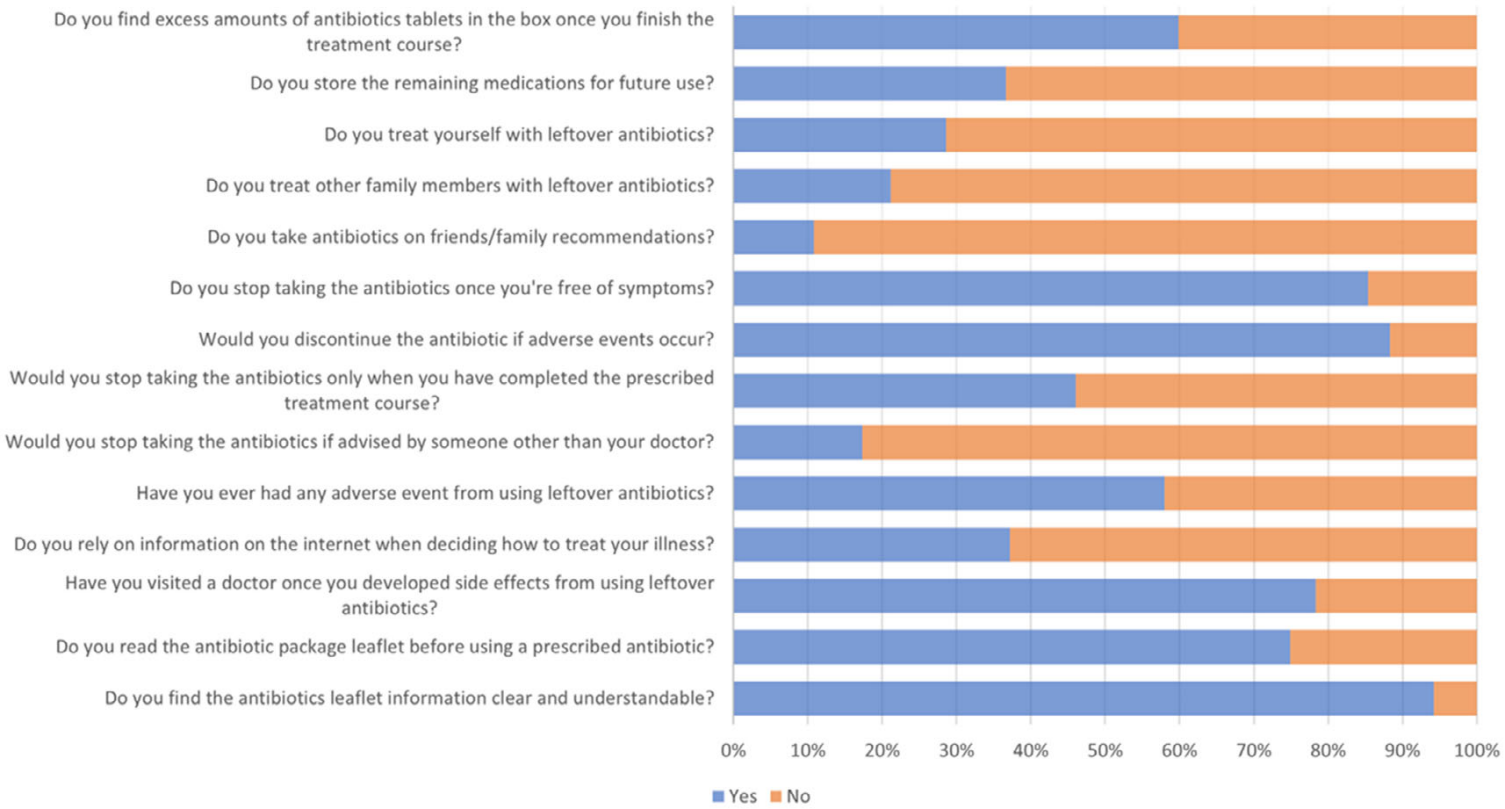
Knowledge about the Antibiotics.

There is no correlation between average household income and the storage and reuse of antibiotics (p>0.30). A statistically significant correlation existed between age and the use of unused antibiotics (p=0.0019). The remaining 311 participants did not use any leftover antibiotics: 33 (22.4%) were under 20 years of age, 77 (52.38%) were 20—29 years of age, 18 (12.24%) were 30—40 years of age, and 19 (12.93%) were older than 40 years of age. There was no statistically significant correlation between gender and the use of leftover antibiotics (p>0.05); among the 147 participants who used leftover antibiotics, 79 were male (53.74%) and 68 were female (46.26%). There was a statistically significant correlation between having children and reusing antibiotics; among the 147 participants who reused antibiotics, 30 (20.41%) had children, while 117 (79.59%) did not. There was no statistically significant correlation between the number of children and the use of leftover antibiotics. Eight (26.67%) of the 30 participants who used leftover antibiotics and had one or two children, nine (30%) had three or four children, and 13 (43.33%) had more than four children. There was no statistically significant correlation between the level of education and the use of unused antibiotics. Among the 147 participants who used leftover antibiotics, 102 (69.39%) had a college degree or higher, while 45 (30.61%) did not complete high school. There was no statistically significant correlation between occupation and the use of leftover antibiotics; 45 (30.61%) of the 147 participants who used leftover antibiotics were healthcare workers, while 102 (69.39%) were non-healthcare workers. We also found no statistical significance between chronic illness and knowledge of antibiotics or attitude toward unused antibiotics. Additional answers are depicted in Figure 1.

## Discussion

When asked what antibiotics are and when they should be used, we found that having 738 participants from all regions of Saudi Arabia helped us better understand the participants’ various backgrounds and educational levels. Providing an analysis of the impact of media and awareness campaigns, when asked when they typically begin using antibiotics, 95.66% (n=706) of respondents said after consulting with a physician. This majority may indicate a high level of awareness, but it may also suggest that the health ministry is effectively implementing and monitoring the restrictive system. Another study in Saudi Arabia in 2021 revealed that 82% of participants who could obtain antibiotics did so without a prescription. ^13^ One reason for this high percentage is that the study was conducted among healthcare students. The studies conducted before the MOH restriction on antibiotics showed a much higher percentage of antibiotic usage without a prescription. ^5^^,^^14^^,^^15^

In our study, most participants took antibiotics after consulting a physician, while others practised self-medication with antibiotics. On the other hand, a study conducted in Kuwait revealed that 27.5% of the study population had taken antibiotics without a prescription. Most (51.9%) of the self-medicated respondents reported giving an antibiotic to another individual. In Kuwait, 59 (31.6%) of self-medicated respondents purchased antibiotics directly from private pharmacies. ^4^ Likewise, in another study, most of those surveyed (92%) acknowledged using antibiotics that weren’t prescribed after receiving advice from a pharmacist. ^16^ Antibiotics were provided without a prescription in 77.6% of cases, and the patient’s request in 95% of cases, according to a study that polled 327 pharmacists. ^17^ Despite Saudi Arabian government legislation stating that antibiotics can only be prescribed by doctors, pharmacies and the general people often disregard this. ^18^

A study revealed that 81.4% of the respondents believed that antibiotic resistance occurs when the body develops a resistance to antibiotics and ceases to function properly. ^19^ When asked what they believe could be done to decrease antibiotic resistance, 88.08% (n=650) of respondents believe that antibiotic campaigns can alter community habits. Community-wide attitude shifts can be easily implemented with campaigns, resulting in a decline in antibiotic resistance. There was a statistically significant correlation between participant age and the misuse of antibiotics (p=0.0019). We can attribute the increase in the use of leftover antibiotics in the 20—29 age group to multiple factors, including the high proportion of participants in this age group (51.08%). Numerous perspectives on antibiotic use (n=377) resulted in unregulated use. A recent study conducted in the Aseer region of southern Saudi Arabia revealed that the general populace there lacked understanding about the usage of antibiotics and stressed the significance of raising community awareness while working under the guidance of doctors. ^20^

There was a statistically significant relationship between having children and reusing antibiotics. This statistic indicates that participants with children were more hesitant than those not having any, either because they feared the side effects of reusing antibiotics or because they were more cautious when it came to their children. A 2022 cross-sectional study revealed that 79% of 183 parents were unaware that antibiotics are ineffective against viral illnesses. ^21^

Studies have yielded variable results when addressing the decision to stop antibiotics once the patient is symptom-free; however, 71.1% of patients did not complete their antibiotic treatment because they felt better. ^14^ A 2018 survey revealed that 11.6% of respondents believed antibiotics should be discontinued as soon as symptoms subside. While our research yielded similar results to the first study, with 71.41% (n=527) of respondents stating that they completed the course as instructed, the first study had a larger sample size. ^22^ The presence of expired pharmaceuticals in the home can increase the risk of toxicity, suicide, and accidental poisoning of children. ^19^ In contrast, unused antibiotics indicate antibiotic misuse and may increase the likelihood of antibiotic resistance. ^23^

A study determined the prevalence of antibiotic self-medication in Saudi Arabia. Over one-third of respondents, or 43.4% reported that they self-medicate with antibiotics on occasion. ^5^ While our study demonstrates that the majority of participants only used antibiotics when prescribed, some participants used antibiotics off-label. Tonsillitis and pharyngitis account for 76.7% of all instances of antibiotic self-medication. ^5^ Our study reveals that 38.08% of individuals who self-medicate with leftover antibiotics do so for sore throat, 32.11% for fever, 27.10% for the common cold, and 2.71% for cough. Another study assessed the knowledge and attitudes of the general population in Jeddah, Saudi Arabia, regarding the use of antibiotics. Nearly half of antibiotic users obtained them without a prescription from a retail pharmacy (63.9%), a private clinic (15.3%), or someone else’s supply (20%). This study was published in 2018, before the 2019 announcement of penalties for violating Saudi Arabia’s restrictive regulations. ^10^ The regulations, announced for the first time in 2015, stipulated that pharmacists who sell antibiotics without a written prescription will be subject to a 100,000 SAR fine, license revocation, or six months in jail. In addition, they discovered that fever, pain, or inflammation were the most common reasons for taking antibiotics (58.2%), followed by respiratory illnesses (21.2%). ^15^ In a study conducted in Saudi Arabia to evaluate the knowledge of health science students regarding antibiotics, 50% of participants believed antibiotics could be used without consulting a doctor. ^13^ Most of the participants in our study believed antibiotics should only be used when prescribed, with 48% (n=204) of these respondents being healthcare professionals.

## Conclusions

The study indicated that nearly half of the participants used leftover antibiotics. Participants having children in their homes significantly reuse antibiotics. However, one-third of the participants didn’t complete the antibiotics course. While it is understandable that people may believe storing leftover antibiotics will be useful in the event of an emergency, there are numerous risks associated with doing so. Although the Saudi Ministry of Health has implemented stringent measures to reduce antimicrobial resistance caused by antibiotic misuse by restricting the dispensing of antibiotics from pharmacies without a prescription, a sizeable proportion (20%) of our sample participants, regardless of age, level of education, or profession, have continued to store leftover antibiotic pills after an infection treatment and reuse them. This calls for further revision of the current measures to fill those gaps and reduce this practice, such as selling/distributing the exact number of pills required to treat the current infection per prescription and increasing awareness of the consequences of using antibiotics excessively without consulting a doctor. This may also have an economic benefit, as more antibiotic pills will be available for additional prescriptions.

## Ethical considerations

Ethical approval was received from the institutional review board of The Majmaah University for research committee (MUREC) (HA-01-R-088) with the ethical number MUREC-f uly.28/COM-2022/1O-2. Information from the questionnaire will be kept confidential and only used for statistical purposes.

## Data Availability

Due to confidentiality of our participants and our university ethical consideration, our data cannot be shared widely/openly. The datasets generated during and/or analyzed during the current study are not publicly available, but are available from the corresponding author on reasonable request after an Ethical Committee approval (Name: Bader Almehmadi, Assistant professor of medicine, rheumatology consultant; email: b.almehmadi@mu.edu.sa; Article type Research article). The access to the data can be shared/granted for research process only and after the approval involving ethics committee and corresponding author. Figshare: Influence of leftover antibiotics on self-medication in Saudi Arabia أثر بقايا المضادات الحيوية في المنازل على سلوك المعالجة الذاتية في المملكة العربية السعودية .pdf. https://doi.org/10.6084/m9.figshare.22001894.v1 ^12^ This project contains the following extended data:
• Influence of leftover antibiotics on self-medication in Saudi Arabia أثر بقايا المضادات الحيوية في المنازل على سلوك المعالجة الذاتية في المملكة العربية السعودية .pdf (Questionnaire). Figshare: STROBE checklist for ‘Influence of leftover antibiotics on self-medication in Saudi Arabia: a cross-sectional study’. https://doi.org/10.6084/m9.figshare.22001603.v1. ^11^ Data are available under the terms of the Creative Commons Attribution 4.0 International license (CC-BY 4.0).
