# Supplementary material for: Knowledge and Attitude of self-medication with leftover antibiotics in Saudi Arabia: A cross-sectional study
Source: F1000Res. 2024 Oct 18;12:304. Originally published 2023 Mar 20. [Version 2] doi: 10.12688/f1000research.130364.2 (PMC11607477; doi:10.12688/f1000research.130364.2)
Supplement: Peer review history

# Reviewer report: Reviewer response for version 2

**DOI:** 10.5256/f1000research.173086.r333403

**Published:** 2024-11-29

**Authors:** Anas Ali Alhur

**Affiliations:** 1 College of Public Health and Health Informatics, University of Hail, Hail, Hail Province, Saudi Arabia

1- Is the work clearly and accurately presented and does it cite the current literature?

 Yes, based on my observations all other it current and recent citations used and strongly align with the paper. 

 2- Is the study design appropriate and is the work technically sound?

  Yes, this designed used for the paper is very good to study nature.

 3- Are sufficient details of methods and analysis provided to allow replication by others?

 No, they should provide more info regarding the instance methods the distribution terms, and the names of questionnaire items.

 4-If applicable, is the statistical analysis and its interpretation appropriate?

 Partly, because no advanced analysis was incorporated to enrich the findings.

 Are all the source data underlying the results available to ensure full reproducibility?

 Yes

 Are the conclusions drawn adequately supported by the results?

 Yes, BUT:

 There is room for improvement, and you should avoid reporting the statistics. The study's limitations and future dictions (recommendations) needed to be discussed.

Is the work clearly and accurately presented and does it cite the current literature?

Yes

If applicable, is the statistical analysis and its interpretation appropriate?

Partly

Are all the source data underlying the results available to ensure full reproducibility?

Partly

Is the study design appropriate and is the work technically sound?

Yes

Are the conclusions drawn adequately supported by the results?

Yes

Are sufficient details of methods and analysis provided to allow replication by others?

No

Reviewer Expertise:

Public Health, Health Informatics, Health Information Management, Information seeeking behaviours

I confirm that I have read this submission and believe that I have an appropriate level of expertise to confirm that it is of an acceptable scientific standard, however I have significant reservations, as outlined above.

## References

[1] : Knowledge and Attitude of self-medication with leftover antibiotics in Saudi Arabia: A cross-sectional study. *F1000Research* .2024;12: 10.12688/f1000research.130364.2 10.12688/f1000research.130364.2 PMC1160747739618514

# Reviewer report: Reviewer response for version 2

**DOI:** 10.5256/f1000research.173086.r332959

**Published:** 2024-10-31

**Authors:** Vitus Silago

**Affiliations:** 1 Department of Microbiology and Immunology, Weill Bugando School of Medicine, Catholic University of Health and Allied Sciences-Bugando, Mwanza, Tanzania

The authors have sufficiently addressed my comments. I recommend this manuscript for indexed.

Is the work clearly and accurately presented and does it cite the current literature?

Partly

If applicable, is the statistical analysis and its interpretation appropriate?

Yes

Are all the source data underlying the results available to ensure full reproducibility?

Yes

Is the study design appropriate and is the work technically sound?

Yes

Are the conclusions drawn adequately supported by the results?

Partly

Are sufficient details of methods and analysis provided to allow replication by others?

Partly

Reviewer Expertise:

NA

I confirm that I have read this submission and believe that I have an appropriate level of expertise to confirm that it is of an acceptable scientific standard.

# Reviewer report: Reviewer response for version 1

**DOI:** 10.5256/f1000research.143118.r219360

**Published:** 2023-11-28

**Authors:** Vitus Silago

**Affiliations:** 1 Department of Microbiology and Immunology, Weill Bugando School of Medicine, Catholic University of Health and Allied Sciences-Bugando, Mwanza, Tanzania

Abstract

 The conclusion does not reflect what the authors investigated, for instance, the authors investigated individual behavior on how they use antibiotics they were prescribed and not that dispensers at community pharmacies are dispensing without prescriptions. I recommend rephrasing this section to reflect what was done and recommend based on specific findings. The interesting finding is people are not completing their doses, they are misusing antibiotics (one among main drivers of antimicrobial resistance). Therefore, authors may recommend something on proper antibiotics use at community levels.

 Introduction

 The first paragraph: …..the misuse of unused antibiotics…., what did Authors mean by “unused” antibiotics? If they meant for “left-over” antibiotics then the term (unused antibiotics) is not proper and if the term (unused) antibiotics are not for “left-over” it is important that the Authors define what they mean by “unused antibiotics”

 “Left-over” antibiotic indicates that an individual did not complete the full course and the next individual, who may be the same or different person to use the “left-over” antibiotic, will not complete the full course as well. Therefore, from this general knowledge, Authors should add one or two sentences explaining how bacteria develops (or acquires) resistance from incomplete course or left-over

 I suggest that Authors shorten the Introduction section particularly by referring few and up-to-date studies on “left-over” and “self-medication”

 The last paragraph of Introduction section should cover gap, problem statement and main objective of the study.

 Methods

 What social media were used to distribute online questionnaires?

 How many questionnaires were returned and how many passed to final data analysis after quality check? What were the reasons (e.g., incompleteness, age limits, e.t.c) for dropping out some responses/questionnaires?

 The last sentence of “Target population” section is not important, it can be deleted

 “Patient consent” and “Instrument of data collection” section may be merged together because they present more similar information

 Results

 Authors should avoid starting a sentence with numerals

 When asked when they begin using antibiotics,

 ……95.66% (n=706) of the participants responded, after consulting a physician, 3.25 % (n=24) said when they felt ill for any reason, and 1.08% (n=8) replied after first attempting herbal medicine. This sentence is not clear, those 3.25% and 1.08% practiced self-medication with reasons of onset of illness and after failed herbal treatment?

 Refer to this sentence “71.41% (n=527) of participants responded with strong agreement to the question, 21% (n=155) with agreement, 4.61% (n=34) with a neutral response, 2.44% (n=18) with disagreement, and 0.54% (n=4) with strong disagreement”, which question did Authors referred to?

 Paragraph 4 (last paragraph of Results section): it is important to perform sub-analysis (if data is available) on the proportion of participants with left-over antibiotics at their homes. This will highlight on the burden of incomplete antibiotic use (and most important the risk of using left-overs) among studied population before reporting factors driving for incomplete antibiotic use

 Discussion

 First paragraph, last sentence: a study from 2021 is contradicting with the findings of the current study and Authors should clearly state this. The previous study shows that 82% of participants obtained antibiotics without prescriptions indicating poor practice among community dispensers (most likely) while the current study shows that 95.66% of respondents obtained antibiotics after medical consultation hence with prescriptions. What could be the possible reason(s) if any for this significant change?

 Second paragraph, second sentence: Authors wrote that, “97 (51.9%) of the self-medicated respondents reported giving an antibiotic to another individual without a prescription”. For my opinion, if an individual self-medicate him/herself, use incomplete course, and then hand-over the left-over antibiotic to the next person it is obvious that no prescription will be required or provided. Therefore, it is not important to end the sentence with “without a prescription”.

 General comment on Discussion section: Authors kept on repeating their results instead of discussing them.

 Conclusion

 It should be revised. For example, measures implemented by the Ministry of Health (Saudi Arabia) notably limiting community access to antibiotics from community pharmacies without prescription has nothing to do with people’s behavior of using incomplete antibiotic use and storing them for future use. Moreover, it should be noted that it is not “excess” antibiotics (excess sounds like they were prescribed extra antibiotics from actual course they were supposed to be prescribed), this needs correction.

Is the work clearly and accurately presented and does it cite the current literature?

Partly

If applicable, is the statistical analysis and its interpretation appropriate?

Yes

Are all the source data underlying the results available to ensure full reproducibility?

Yes

Is the study design appropriate and is the work technically sound?

Yes

Are the conclusions drawn adequately supported by the results?

Partly

Are sufficient details of methods and analysis provided to allow replication by others?

Partly

Reviewer Expertise:

Antimicrobial Resistance and Molecular Epidemiology of Infectious Diseases

I confirm that I have read this submission and believe that I have an appropriate level of expertise to confirm that it is of an acceptable scientific standard, however I have significant reservations, as outlined above.

# Response

**Published:** 2024-10-06

**Authors:** Bader Almehmadi

**Affiliations:** College of Medicine, Majmaah University, Al-Majmaah, 11952, Saudi Arabia, Saudi Arabia

Dear Reviewer, 

 Thank you for the suggestion, changes have been done accordingly throughout the manuscript.

# Reviewer report: Reviewer response for version 1

**DOI:** 10.5256/f1000research.143118.r209852

**Published:** 2023-11-27

**Authors:** Mohamad Al-Tannir

**Affiliations:** 1 Applied Clinical Research Administration, Research Center, King Fahad Medical City Saudi Arabia, Riyadh, Saudi Arabia

1. Title and objective do not perfectly align.
2. The Introduction is written as a literature review of a proposal rather than an introduction of a manuscript.
3. Methods section lacks important information about the questionnaire and its validity, and the sample size seems underestimated. IRB name and Log should be provided.
4. Results section is written inappropriately.
5. Discussion section should follow the rule of 7 paragraphs of a discussion of a scientific manuscript.
6. English proofreading is a must.

Is the work clearly and accurately presented and does it cite the current literature?

Partly

If applicable, is the statistical analysis and its interpretation appropriate?

Partly

Are all the source data underlying the results available to ensure full reproducibility?

Partly

Is the study design appropriate and is the work technically sound?

Partly

Are the conclusions drawn adequately supported by the results?

No

Are sufficient details of methods and analysis provided to allow replication by others?

No

Reviewer Expertise:

Public health, multidisciplinary clinical research and clinical trials.

I confirm that I have read this submission and believe that I have an appropriate level of expertise to confirm that it is of an acceptable scientific standard, however I have significant reservations, as outlined above.

# Response

**Published:** 2024-10-06

**Authors:** Bader Almehmadi

**Affiliations:** College of Medicine, Majmaah University, Al-Majmaah, 11952, Saudi Arabia, Saudi Arabia

1. Title and objective do not perfectly align.
**response: changes have been made**
2. The Introduction is written as a literature review of a proposal rather than an introduction of a manuscript.
   
**response: Introduction has been thoroughly edited as per the reviewer suggestions**
3. Methods section lacks important information about the questionnaire and its validity, and the sample size seems underestimated. IRB name and Log should be provided.
**response: changes have been made. IRB details and log is provided in the end of study.**
4. Results section is written inappropriately.
**response:  changes have been made**
5. Discussion section should follow the rule of 7 paragraphs of a discussion of a scientific manuscript.
   
**response:  changes have been made**
6. English proofreading is a must. **response:  changes have been made**

# Reviewer report: Reviewer response for version 1

**DOI:** 10.5256/f1000research.143118.r205470

**Published:** 2023-09-29

**Authors:** Juan Manuel Vazquez-Lago

**Affiliations:** 1 Health Research Institute of Santiago de Compostela (IDIS) & Service of Preventive Medicine and Public Health, Clinic Hospital of Santiago de Compostela, Santiago de Compostela, Spain

Thank you very much for allowing me to review this work. It is an interesting work, focused on a major public health problem, the use of antibiotics. However, the article, from my point of view, needs improvement to be published in this journal.

 Formal aspects: The text does not present line numbering, which makes it difficult to prepare a review report.

 Abstract:

1. Methods: The methods reflected in the abstract are scarce. Is the questionnaire used a validated questionnaire?
2.  Results: Part of the results are methodology. "from all ages...of Saudi Arabia", since they are inclusion criteria (they do not contribute anything to the results section. The response rate to the questionnaire, the distribution of the sample in terms of sex and age (mean and SD). Where "147 participants..." is reported, it does not provide much information to the results (a relative value is better).
3. Conclusions: The conclusions expressed in the abstract are not supported by the results. It must be rewritten.

Introduction:

1. It must be reduced. It is difficult to read. The introduction should be structured in 3 paragraphs: (1) describe the topic within which the text is framed; (2) report the gap or lack of knowledge that the text attempts to resolve; (3) propose the questions or objectives that guide the text

Methodology: this section is very brief. It should be improved.

1. The questionnaire is not provided. It should be contributed to be able to value it. Has this questionnaire been validated on the target population? If not validated, all results are largely compromised. This possible weakness should also be discussed.
2. Has the questionnaire only been distributed through social networks? People who do not use social networks did not have the opportunity to complete the questionnaire? This is a very important selection bias, as well as an information bias, since access to social networks can be influenced, for example, by social class, educational level, etc., sex...
3. It is not necessary to include the sample size calculation formula in the text.

Results:

1. It should be stated how many were sent the questionnaire and how many of them responded. At no time is the response rate reported.
2. The distribution by sex and age must always be reported in the text.

Discussion:

1. Poorly structured. It should be reviewed.
2. In the first paragraph the most notable result of the investigation should always be reported. What does this research contribute new to the scientific world?
3. There are no references, or at least I have not been able to objectify them, to the limitations and strengths of the design.
4. You can discuss the results obtained with other recently published articles, in order to increase the external validity of your work. (Antibiotics (Basel). 2023 Mar 11;12(3):558. doi: 10.3390/antibiotics12030558.) (Antibiotics (Basel). 2023 Feb 24;12(3):457. doi: 10.3390/antibiotics12030457.)

Conclusions:

1. The paragraph that refers to the conclusions is more a part of the discussion. Conclusions should be written in 2-3 short sentences strongly supported by the results.

Is the work clearly and accurately presented and does it cite the current literature?

Partly

If applicable, is the statistical analysis and its interpretation appropriate?

Partly

Are all the source data underlying the results available to ensure full reproducibility?

No

Is the study design appropriate and is the work technically sound?

Partly

Are the conclusions drawn adequately supported by the results?

Partly

Are sufficient details of methods and analysis provided to allow replication by others?

No

Reviewer Expertise:

Antibiotics

I confirm that I have read this submission and believe that I have an appropriate level of expertise to state that I do not consider it to be of an acceptable scientific standard, for reasons outlined above.

## References

[1] : Knowledge, Perceptions, and Perspectives of Medical Students Regarding the Use of Antibiotics and Antibiotic Resistance: A Qualitative Research in Galicia, Spain. *Antibiotics (Basel)* .2023;12(3) : 10.3390/antibiotics12030558 10.3390/antibiotics12030558 36978424 PMC10044116

[2] : Knowledge, Attitudes and Practice Regarding Antibiotic Prescription by Medical Interns: A Qualitative Study in Spain. *Antibiotics (Basel)* .2023;12(3) : 10.3390/antibiotics12030457 10.3390/antibiotics12030457 36978321 PMC10044181
